# Neutrophils: Beneficial and Harmful Cells in Septic Arthritis

**DOI:** 10.3390/ijms19020468

**Published:** 2018-02-05

**Authors:** Daiane Boff, Helena Crijns, Mauro M. Teixeira, Flavio A. Amaral, Paul Proost

**Affiliations:** 1 Imunofarmacologia, Department of Biochemistry and Immunology, Instituto de Ciências Biológicas, Universidade Federal de Minas Gerais, Belo Horizonte 31270-901, Brazil; daiane.bff@hotmail.com (D.B.); helena.crijns@kuleuven.be (H.C.); mmtex@gmail.com (M.M.T.); dr.famaral@gmail.com (F.A.A.); 2 Laboratory of Molecular Immunology, Department of Microbiology and Immunology, Rega Institute for Medical Research, KU Leuven, B-3000 Leuven, Belgium

**Keywords:** neutrophil, septic arthritis, chemoattractant, *Staphylococcus aureus*, tissue damage, infection

## Abstract

Septic arthritis is an inflammatory joint disease that is induced by pathogens such as *Staphylococcus aureus*. Infection of the joint triggers an acute inflammatory response directed by inflammatory mediators including microbial danger signals and cytokines and is accompanied by an influx of leukocytes. The recruitment of these inflammatory cells depends on gradients of chemoattractants including formylated peptides from the infectious agent or dying cells, host-derived leukotrienes, complement proteins and chemokines. Neutrophils are of major importance and play a dual role in the pathogenesis of septic arthritis. On the one hand, these leukocytes are indispensable in the first-line defense to kill invading pathogens in the early stage of disease. However, on the other hand, neutrophils act as mediators of tissue destruction. Since the elimination of inflammatory neutrophils from the site of inflammation is a prerequisite for resolution of the acute inflammatory response, the prolonged stay of these leukocytes at the inflammatory site can lead to irreversible damage to the infected joint, which is known as an important complication in septic arthritis patients. Thus, timely reduction of the recruitment of inflammatory neutrophils to infected joints may be an efficient therapy to reduce tissue damage in septic arthritis.

## 1. Introduction

**Figure 1:**
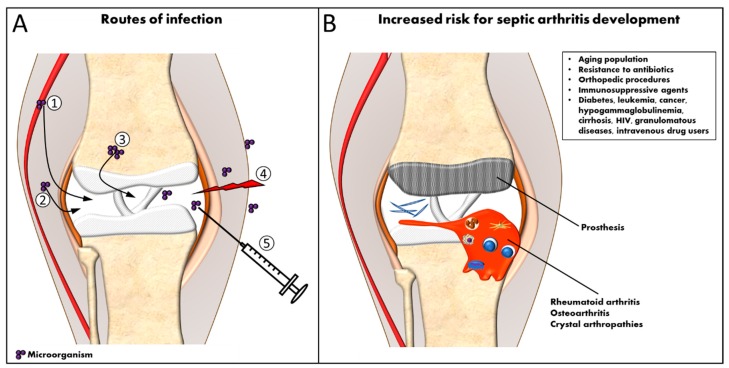
Routes of bacterial infection and risk factors for septic arthritis development. (**A**) Bacteria can access the joint through 5 routes: (1) by hematogenous spread; (2) from an adjacent infected tissue; (3) through infected bones; (4) as a consequence of trauma or (5) during diagnostic procedures. (**B**) Additionally, some risk factors are related to septic arthritis such as presence of other rheumatic or immunosuppressive diseases, prosthetic surgery and higher age.

Septic arthritis can be defined as an inflammatory disease of the joints, induced by an infectious agent [1,2]. Bacteria, viruses, fungi and protozoa may invade joints and cause injury. However, Gram-positive bacteria, especially *Staphylococcus aureus* (*S. aureus*), are the most prevalent microorganisms causing septic arthritis [3]. In addition, *S. aureus* is responsible for the most severe cases of septic arthritis. Any synovial joint can be involved; however, most frequently one large joint such as the knee or hip is affected [1,4]. Invasion of bacteria into the synovial space can occur predominantly by two routes: either through hematogenous spread (most common) or by direct invasion [5] as shown in Figure 1A. The synovium is extremely vascularized and contains no limiting basement membrane, facilitating the access to the synovial space. Thus, bacteria may spread directly from adjacent osteomyelitis or from a local soft tissue infection and could reach the joint during diagnostic or therapeutic procedures, penetrating trauma, or prosthetic surgery, or, less commonly, by animal bites [2,6,7].

Septic arthritis patients typically present with a single swollen, warm and painful joint with a decreased range of motion. Fever is present in only 30–40% of cases [8]. Normally a single synovial joint is affected such as the knee, hip, ankle or elbow. The hip is the more frequently affected joint in children. Atypical joint infection, including the sternoclavicular, costochondral and sacroiliac joints, may be common in intravenous drug users [9]. Polyarticular septic arthritis is not common and usually accompanied by a number of risk factors. The articular damage is an important feature and a challenge in this disease, since about 25–50% of patients have irreversible articular damage with total loss of joint function [1,10].

Once microorganisms have gained entry into the joint, the low fluid shear conditions in the joint space allow adherence and infection. The attachment of *S. aureus* to the joint extracellular matrix or to implanted medical devices, such as prosthetic joints, is mediated by microbial surface component recognizing adhesive matrix molecules (MSCRAMMs). After colonizing the joint, the bacteria can rapidly proliferate and trigger an acute inflammatory response [11]. The synovium responds with a proliferative lining-cell hyperplasia and there is an influx of inflammatory cells [12]. Phagocytes, including neutrophils and macrophages, chemotactically migrate to the infected joint, directed by gradients of bacterial products displaying chemotactic activity and mediators of the immune response [13]. Neutrophils play a major role in the first-line defense against invading pathogens, including bacteria, and these leukocytes are the first to migrate to the site of infection. Activated macrophages are recruited to the joint slightly later and they are followed by T lymphocytes [1,14,15,16]. In this manuscript, we will review current knowledge on septic arthritis with an emphasis on the role of neutrophils. We will discuss neutrophil activation and recruitment, not only resulting in their beneficial role in the elimination of microbial infection, but also regularly causing tissue damage and permanent joint dysfunction.

## 2. Septic Arthritis

Septic arthritis is an uncommon pathology of which the yearly incidence is estimated to be 3 to 12 cases per 100,000 people in industrialized countries [17,18,19,20]. Septic arthritis can affect people at any age, but elderly people and very young children are more frequently affected [21,22]. Approximately half of the patients are younger than three years and one-third are under the age of two. The incidence is low in children younger than three months. Furthermore, males are slightly more susceptible than females [2,23]. The incidence of septic arthritis appears to decrease in children in the United States [23]. However, several factors including an ageing population, a growing resistance to antibiotics, an increase in infections related to orthopedic procedures and an enhanced use of immune modulating agents, contribute to an increase of septic arthritis in the general population [7,24,25,26]. Furthermore, the presence of previous joint diseases, such as rheumatoid arthritis (RA), osteoarthritis, crystal arthropathies and other forms of inflammatory arthritis is a predisposing factor for the development of infectious arthritis (Figure 1B). In particular, the incidence of septic arthritis is approximately 10-fold higher in patients with RA, in comparison to the general population [27,28]. The incidence of septic arthritis increases not only in previous arthritic patients, but also in people who suffer from other chronic and immunosuppressive diseases, such as diabetes, leukemia, cirrhosis, granulomatous diseases, cancer, hypogammaglobulinemia, human immunodeficiency virus (HIV)-infected patients and intravenous drug users [29,30,31]. Hemodialysis has been reported as an important risk factor for septic arthritis [32]. Also, penetrating trauma, including animal bites and local therapeutic intra-articular corticosteroid injections may cause septic arthritis in atypical joints [11,33,34]. Recent joint surgery is also associated with an increased risk of infection [35,36]. In addition, several cases of joint infections have been reported in patients that received immunosuppressive therapy and/or glucocorticoids [37]. In this context, the use of classic disease modifying anti-rheumatic drugs (DMARDs) in RA patients can be an additional risk factor that facilitates the development of infectious arthritis [38,39]. Although data from observational registers have suggested an increased incidence of joint infections in patients receiving anti-tumor necrosis factor (TNF) therapy, the incidence does not seem to be different from the incidence in patients treated with classical DMARDs [40].

Septic arthritis is associated with significant mortality and morbidity. Moreover, it is a rheumatologic emergency, since irreversible joint destruction and consequently loss of function of the joint can occur rapidly [24,25,41]. Septic arthritis has a mortality ranging from approximately 10% to, depending on the report, more than 50% in case of polyarticular disease [3,7,41]. Persisting joint damage occurs in more than 30% of the patients [41]. Early diagnosis and immediate and effective treatment are essential to prevent severe outcomes such as irreversible joint destruction or death [2,23]. Furthermore, the general state of the patient and the number, type and resistance pattern of the causing agent are also of significance to the outcome [42].

The most common causative agent associated with septic arthritis is *S. aureus*, which accounts for about 50% of cases [43]. Recently, an increase in methicillin-resistant *S. aureus* (MRSA) infections has been reported in several health-care systems, particularly in the elderly and intravenous drug abuser populations as well as in patients who underwent orthopedic procedures [44]. MRSA has been associated with 18% and 41% of septic arthritis cases in studies in São Paulo, Brazil and Tainan, Taiwan, respectively [44,45]. Other bacteria such as group *B* streptococci, *Streptococcus pneumoniae*, *Neisseria gonorhoeae*, *Pseudomonas aeruginosa*, *Escherichia coli*, *Proteus* genus and *Klebsiella* species can be associated with septic arthritis, but are less frequent [46]. Common causative agents in children include *S. aureus*, *Streptococcus pneumonia* and *Kingella kingae* [47]. The infectious capacity of *S. aureus* in different tissues is provided by the presence of several virulence factors [48].

*S. aureus* has a capsule composed of polysaccharides, which acts as a physical barrier that protects the bacteria from phagocytosis by immune cells [49]. Peptidoglycan (PGN) is the major component of the cell wall of Gram-positive bacteria. Bacterial PGN was detected in synovial tissue of patients with septic arthritis [50] and studies demonstrated that intra-articular injection of PGN in mice can cause arthritis [51]. *S. aureus* is a bone pathogen because it possesses several cell-surface adhesion molecules that facilitate its binding to the bone matrix [52]. Binding involves a family of adhesins that interact with extracellular matrix components and these adhesins have been termed MSCRAMMs [53]. Specific MSCRAMMs are needed for the colonization of specific tissues. Particular MSCRAMMs include fibronectin-binding proteins, fibrinogen-binding proteins, elastin-binding and collagen-binding adhesion molecules. Once the bacteria adhere to and colonize bone matrix, they elaborate several virulence factors such as proteases, which can break down matrix components [54]. Further experimental studies demonstrated that collagen adhesin is an important virulence determinant in *S. aureus*-induced arthritis [55].

*S. aureus* secretes a large number of enzymes and toxins, many of which have been implicated as potential virulence factors. Alpha and gamma toxins are lytic to red blood cells and various leukocytes, but not to neutrophils [56]. The combination of these two toxins has been experimentally demonstrated to be important for the development of septic arthritis [57]. Another toxin is Panton–Valentine leukocidin (PVL, consisting of the LukS and LukF proteins) that can lyse leukocytes, especially human neutrophils, and is related to fulminant cases of septic arthritis [58]. Enterotoxins, such as the superantigen toxic shock syndrome toxin-1 (TSST-1) can cause shock by stimulating the release of interleukin (IL)-1, IL-2, TNF and other cytokines [59]. Experimentally, the presence of TSST-1 favors the development of septic arthritis [60]. Another important virulence factor is bacterial deoxyribonucleic acid (DNA) with non-methylated CpG motifs, which is considerably less frequent in vertebrate DNA [61]. The CpG DNA can bind to Toll-like receptor 9 (TLR9) in immune cells, leading to the production of cytokines such as IL-1β, TNF, IL-6 and IL-12 [62,63]. Some studies showed that intra-articular injection of *S. aureus* CpG DNA can induce arthritis in mice [64,65].

## 3. Diagnosis and Treatment of Septic Arthritis

Gram staining and cultures of synovial fluid should be investigated in any case of suspected septic arthritis. Antibiotic therapy is started ideally after synovial fluid samples have been obtained [64]. Gram stains of synovial fluid are helpful when positive, but they are not always sensitive enough for the diagnosis of septic arthritis [65]. Patients should be treated empirically for septic arthritis when synovial fluid leukocyte counts exceed 50,000 cells/mm^3^, although gout and pseudogout also commonly present with leukocyte counts of this magnitude [66]. Thus, the analysis of the presence of urate crystals in synovial fluid by polarized light microscopy is very important for the exclusion of a gouty attack [67,68,69]. Furthermore, the analysis of the delta neutrophil index (DNI) could be a valuable tool to distinguish septic arthritis and gout. DNI is a value that corresponds to the fraction of circulating immature granulocytes, reflecting a burden of infection. In this context, a study demonstrated that septic arthritic patients presented with a significantly higher DNI as compared to acute gouty attack patients, suggesting DNI as complementary predicting tool for septic arthritis diagnosis [70]. However, the serum procalcitonin level also appears to be a promising marker for septic arthritis [71]. On the other hand, mono-arthritis can also be misdiagnosed with cases of SAPHO (Synovitis-acne-pustulosis-hyperostosis-osteitis) syndrome, characterized by a combination of skin and osteoarticular manifestations [72]. Although *S. aureus* and other pathogens have been isolated from affected tissues [73], radiology features, such as radiography and MRI mainly in sternoclavicular joints are necessary for SAPHO syndrome diagnosis, especially in the absence of dermatological clinical manifestations [72]. Blood cultures should be obtained in all patients with suspected septic arthritis. However, the cultures must be obtained before starting antibiotic treatment to optimize the possibility of isolating the causative bacteria [74]. DNA-based techniques, hybridization probes, polymerase chain reaction (PCR)-based techniques and detection of typical bacterial compounds by mass spectrometry provide quick results [75]. The detection of microorganisms by PCR has shown more accurate results [76]. However, the risk of contamination, the presence of background DNA, the lack of a gold standard and the fact that PCR techniques detect DNA instead of living pathogens make the interpretation of these tests difficult [77].

Imaging can be used as complementary diagnosis since a computed tomography (CT) scan may not depict abnormalities during the early stages of infection. However, CT is a better imaging technique for visualization of local edema, bone erosions, osteitic foci and sclerosis [77]. Magnetic resonance imaging (MRI) provides better resolution for the detection of joint effusion and for differentiation between bone and soft-tissue infections. MRI findings in patients with septic arthritis include joint effusion, cartilage and bone destruction, soft-tissue abscesses, bone edema and cortical interruption [78].

Septic arthritis is so rapidly destructive that broad-spectrum antibiotics are usually warranted until culture data are available or bacteria have been identified by mass spectrometry. Given the increasing importance of MRSA as a cause of septic arthritis, initial antibiotic regimens should generally include an antibiotic active against MRSA, such as vancomycin [79]. Cefazolin is a reasonable alternative in areas with a low prevalence of MRSA. If serious vancomycin allergy is present, empiric therapy utilizing linezolid or daptomycin must be considered [80]. Septic arthritis associated with animal bites should be treated with agents such as ampicillin-sulbactam, which are active against oral microbiota [81].

In general, septic arthritis in adults should be treated for at least 3 weeks, which may include a period of step-down oral therapy [25]. In children with uncomplicated septic arthritis, as few as 10 days of antibiotic therapy may be sufficient [82]. Septic arthritis can be managed with antibiotics combined with joint drainage by arthroscopy, arthrocentesis, or arthrotomy [83,84,85]. Joint drainage decompresses the joint, improves blood flow, and removes bacteria, toxins, and proteases [84]. Arthrocentesis should be repeated daily until effusions resolve and cultures are negative. Aggressive rehabilitation is essential to prevent joint contractures and muscle atrophy [2].

## 4. Immune Response against *S. aureus*

### 4.1. Introduction

Pathogens are controlled by innate and adaptive immune responses and the recognition of microorganisms is the first step in host defense [86]. In the joint, resident cells, such as synoviocytes, can recognize *S. aureus* through pattern recognition receptors (PRRs). In that way, those cells produce inflammatory mediators such as cytokines, chemokines, complement proteins and lipids that will attract neutrophils and macrophages [87]. The complement system plays an important role in host defense against infection. Products of complement activation affect many functions of neutrophils in host defense. The complement system can opsonize microorganisms, thereby stimulating phagocytosis. Phagocytosis of *S. aureus* by neutrophils is of major importance for the outcome in the early stage of septic arthritis. Moreover, chemotaxis of neutrophils to the site of inflammation is facilitated by complement factors such as C5a. Complement depletion, by using cobra venom factor, in a murine model of hematogenously induced *S. aureus* septic arthritis caused an aggravation of septicemia and arthritis [88]. The prevalence and severity of septic arthritis and septicemia-induced mortality were augmented upon complement depletion. Manifestations of the disease, such as synovitis and destruction of cartilage and/or bone, occurred earlier and were more common and severe in the decomplemented mice compared to the control group. Altogether, complement depletion disturbed phagocytosis by impairing opsonization of bacteria, and interfered with the extravasation and migration of neutrophils, leading to a deterioration of the disease [88]. During the onset of the inflammatory process, neutrophils are the main cells recruited to the site of infection and they play a fundamental role in both the phagocytosis and killing of the microorganism [85]. The importance of neutrophils in controlling *S. aureus* in the joint was demonstrated in a study in which neutrophils were depleted. This caused the impairment of bacterial control [15]. Other immune cells such as macrophages [6], natural killer (NK) cells [6] and B lymphocytes [89] are described to have a role in experimental models of septic arthritis. Dendritic cells in *S. aureus*-induced arthritis are fundamental for the activation of the adaptive immune response. The depletion of dendritic cells during *S. aureus* infection in the lungs showed an increase in bacterial load and mortality [90]. During *S. aureus* infection, dendritic cells can induce a Th1 response probably through IL-12 production. Experimentally, the lack of systemic IL-12 increased the bacterial load in the joint during *S. aureus*-induced septic arthritis [91]. Dendritic cells can also stimulate Th17 activation, an important source of IL-17. The cytokine IL-17 has been shown to be important for bacterial clearance and to prevent tissue damage in experimental *S. aureus*-induced arthritis [92].

### 4.2. Neutrophils

Neutrophils are continuously generated in the bone marrow from myeloid precursors. Humans and mice differ in their numbers of circulating neutrophils. In humans, 50–70% of circulating leukocytes are neutrophils, whereas this number drops to only 10–25% in mice [93]. In the circulation, mature neutrophils have a segmented nucleus and their cytoplasm is enriched with granules and secretory vesicles. After the first moments following infection, neutrophils can be recruited from blood vessels to the site of infection, a process that involves a close interaction between neutrophils and endothelial cells and is mediated by different chemotactic agents that activate the cells and guide their migration [94]. Chemotactic factors for neutrophils include bacterial peptides [95], products of complement activation (such as C5a) [96], extracellular matrix degradation products (laminin digests) [97], arachidonic acid metabolites (leukotriene B4/LTB4) [98], other lipid mediators such as platelet activating factors (PAF) [99] and chemokines [100].

Neutrophils are recruited in a cascade of events that involves the following commonly recognized steps that precede the transmigration: tethering, rolling, adhesion, and crawling on the endothelial cell surface [101,102]. Neutrophil recruitment is initiated by changes on endothelial cells during the early steps of inflammation. Endothelial cells can be activated directly by pathogens through PRR activation, causing an increase of the expression and exposure of adhesion molecules on their surface. Once on the endothelial surface, P selectin and E selectin bind to their glycosylated ligands on leukocytes, leading to the tethering (capturing) of free-flowing neutrophils to the surface of the endothelium and subsequent rolling of neutrophils along the vessel in the direction of the blood flow [103]. Rolling requires rapid formation and breakage of adhesive bonds. The rolling of neutrophils facilitates their contact with chemokine-decorated endothelium to induce activation. Full activation may be a two-step process initiated by specific priming by pro-inflammatory cytokines, such as TNF and IL-1β, or by contact with activated endothelial cells followed by an exposure to pathogen-associated molecular patterns (PAMPs), chemoattractants or growth factors [104,105]. The adhesion step of the recruitment cascade prepares neutrophils for transmigration, but migration does not necessarily occur at the initial site of their arrest on the endothelium. Some of the adherent neutrophils reveal so called crawling behavior as they elongate and continue to send out pseudopods, apparently actively scanning and probing the surroundings while remaining firmly attached to a single location within the microvasculature [106]. During the transmigration process, neutrophils cross the endothelium in a process dependent on integrins. The migration across the endothelial cell layer occurs either paracellularly (between endothelial cells) or transcellularly (through an endothelial cell without mixing the cytoplasmic content of both cells). Next, neutrophils migrate towards the infectious/inflammatory focus in the tissue [107].

### 4.3. Neutrophil Functions during Infections

**Figure 2:**
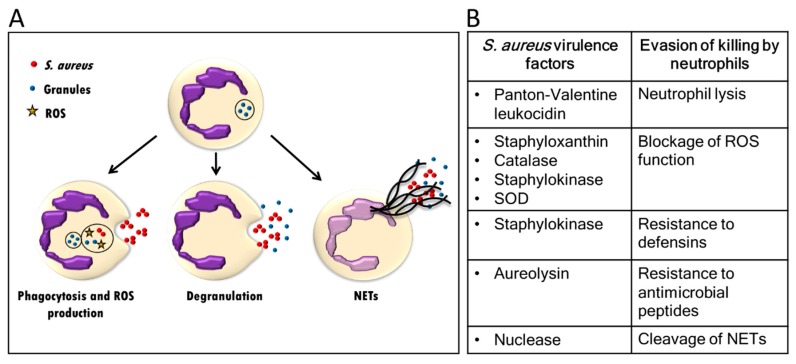
Killing of *S. aureus* by neutrophils and immune evasion. (**A**) Neutrophils phagocytose *S. aureus* and kill the bacteria by the production of ROS, liberation of lytic enzymes from granules and production of NETs. (**B**) *S. aureus* may possess virulence factors including enzymes that kill neutrophils or allow the bacteria to evade killing by neutrophils. ROS: reactive oxygen species; NET: neutrophil extracellular traps; SOK: surface factor promoting resistance to oxidative killing; SOD: superoxide dismutase.

In order to kill microorganisms, neutrophils can phagocyte, secrete the content of their granules, produce reactive oxygen species (ROS) and antimicrobial peptides, and release neutrophil extracellular traps (NETs) as demonstrated in Figure 2A [108]. *S. aureus* may produce several virulence factors that neutralize neutrophil-dependent killing. These include the pore-forming toxin Panton-Valentine leukocidin, antioxidants staphyloxanthin, catalase and superoxide dismutase and the surface factor promoting resistance to oxidative killing (SOK) to neutralize the action of ROS [58,109,110,111] (Figure 2B). Neutrophil defensin-dependent killing of bacteria is inhibited by the binding of neutrophil defensins to staphylokinase [112]. In addition, the neutrophil-derived antibacterial peptide and neutrophil attractant LL37 may be degraded by the *S. aureus* metalloproteinase aureolysin [113,114]. Finally, NETs may be degraded by a *S. aureus* nuclease, resulting in diminished antibacterial efficiency of NETs [115].

Once at the site of infection, the neutrophils bind and ingest invading microorganisms by phagocytosis, a critical first step in the removal of bacteria during infection. Neutrophils recognize numerous surface-bound and freely secreted bacterial products such as PGN, lipoproteins, lipopolysaccharide, CpG-containing DNA, and flagellin [116]. Such conserved bacterial PAMPs are recognized directly by PRRs expressed on the extracellular membrane or on organelles in the cytosol of the neutrophil [117]. The process of neutrophil phagocytosis triggers synthesis of a number of immunomodulatory factors that will recruit additional neutrophils, modulates subsequent neutrophil responses, and coordinates early responses of other cell types such as monocytes, macrophages, dendritic cells and lymphocytes, thereby providing an important link between innate and acquired immune responses [118].

Phagocytosis is accompanied by the generation of microbicidal ROS (oxygen-dependent) and fusion of cytoplasmic granules with microbe-containing phagosomes (degranulation). Degranulation enriches the phagosome lumen with antimicrobial peptides and proteases (oxygen-independent process), which in combination with ROS create an environment non-conducive to survival of the ingested microbe [119]. In the most classical sense, neutrophil activation is intimately linked with the production of superoxide and other secondarily derived ROS, an oxygen-dependent process known as the oxidative or respiratory burst. High levels of superoxide are generated upon full assembly of the multi-subunit nicotinamide adenine dinucleotide phosphate (NADPH)-dependent oxidase in both the plasma- and phagosomal membranes [120,121].

Neutrophils present three fundamental types of granules: primary or azurophilic, secondary or specific and tertiary or gelatinase-containing granules [122,123]. Primary granules are the largest and are formed first during neutrophil maturation. They are named after their ability to take up the basic dye azure A and contain myeloperoxidase (MPO), defensins, lysozyme, bactericidal/permeability-increasing protein (BPI), and a number of serine proteases such as neutrophil elastase, proteinase 3 and cathepsin G [124]. Granules of the second class are smaller, do not contain MPO and are characterized by the presence of the glycoprotein lactoferrin and antimicrobial compounds including neutrophil gelatinase-associated lipocalin, human cationic antimicrobial protein-18 and lysozyme [125]. The gelatinase granules are also MPO-negative, are smaller than specific granules and contain few antimicrobials, but they serve as a storage location for a number of metalloproteases, such as gelatinase and leukolysin [123]. Neutrophils also present secretory vesicles that serve as a reservoir for a number of important membrane-bound molecules active during neutrophil migration. As a neutrophil proceeds through the activation process, granules are mobilized and fuse with either the plasma membrane or the phagosome, releasing their content into the respective environments [126].

Neutrophils produce peptides and proteins that directly or indirectly kill microbes. There are three main types of antimicrobials: cationic peptides and proteins that bind to microbial membranes, enzymes, and proteins that deprive microorganisms of essential nutrients [127]. Many of these peptides disrupt the membrane integrity, whereas some antimicrobials are thought to disrupt essential microbial functions, such as DNA replication, transcription or production of energy [128]. In addition, some of these neutrophil-derived antimicrobial peptides also attract additional leukocytes to the inflammatory site [113,129]. Recently, it was demonstrated that neutrophils can produce neutrophil extracellular traps (NETs) that contain decondensed chromatin, bound histones, azurophilic granule proteins and cytosolic proteins. They have a demonstrated capacity to bind to and kill a variety of pathogens including *S. aureus* [130]. Extrusion of such structures by neutrophils is predicted to limit microbial spread and dissemination, while enhancing effective local concentrations of extruded microbicidal agents, thereby promoting synergistic killing of attached microorganisms [131].

Several mechanisms used by neutrophils to eliminate pathogens can also cause host tissue damage [132]. In that way, recruitment of inflammatory neutrophils needs to be tightly controlled and such neutrophils must be removed before they have serious, detrimental effects on inflamed tissues. Once neutrophils have executed their antimicrobial function, they die via a built-in cell-death program. However, not only does apoptosis reduce the number of neutrophils present, it also produces signals that abrogate further neutrophil recruitment [133]. In addition, evidence is accumulating for the existence of anti-inflammatory neutrophils that produce IL-10 [103]. Indeed, different neutrophil populations were collected from MRSA-resistant versus MRSA-sensitive mice [134]. It is not clear whether these are generated as different populations or evolve separately as a consequence of stimulation with microorganisms, different growth factors or cytokines.

### 4.4. The Chemokine System in Neutrophil Recruitment

Chemokines are small proteins with molecular masses of ~7–12 kDa that belong to the family of chemotactic cytokines. Chemokines are the only group of cytokines that bind to G protein-coupled receptors (GPCRs) [135]. Chemokines were named based on their chemoattractant property, described first in 1987 when CXCL8 was shown to be involved in chemotaxis of neutrophils in vitro [136,137]. Additionally, chemokines were described to be involved in other processes such as embryogenesis, homeostasis, angiogenesis and inflammation [138,139]. Chemokines can be divided into 4 subfamilies based on the position of the two cysteine residues in their N-terminal amino acid sequence: (1) CC chemokines have two adjacent cysteines; (2) CXC chemokines present with one amino acid between the two cysteines; (3) the CX3C chemokine has 3 amino acids between the cysteines, and; (4) C chemokines lack one of the two N-terminal cysteines [140]. The ELR^+^ CXC chemokines that have a specific amino acid sequence of glutamic acid-leucine-arginine (ELR) immediately before the first cysteine of the CXC motif, are associated with neutrophil recruitment and include CXCL1, 2, 3, 5, 6, 7 and 8. Those without an ELR motif rather recruit T and B lymphocytes, monocytes or hematopoietic precursor cells [141,142,143,144,145,146,147].

Chemokines can bind to two types of receptors: GPCRs and atypical chemokine receptors (ACKRs) that do not signal through G proteins and lack chemotactic activity. GPCRs are classified as CCR, CXCR, CX3CR and XCR according to the cysteine motif in their ligands [148,149]. The interactions of human and murine chemokines with GPCRs reported to be expressed on neutrophils are shown in Table 1. CXCR1 and CXCR2 are the abundantly expressed receptors on circulating neutrophils. However, under inflammatory condition, neutrophils in tissues have been reported to express multiple other CXC and CC chemokine receptors including CXCR3, CCR1, CCR2 and CCR3 [150,151]. CXCR4 expression on neutrophils enhances upon aging of neutrophils and has been suggested to be linked to resolution of inflammation [152,153]. As can be seen, one chemokine (e.g., CXCL8) can bind to several receptors and one receptor (e.g., CXCR2) may transduce signals for different ligands. The chemokine interactions that at the first moment were considered as “redundant” gave rise to the term “promiscuity” of the chemokine system. However, much attention is given now to the “bias of the chemokine system”, including ligand bias, receptor bias and tissue bias, which tend to explain and allow us to understand how those chemokines bind to their receptors and promote different responses in different situations [154]. For instance the chemokines CXCL4 and CXCL7 are typical platelet products [155]. In contrast, CCL3, CCL3L1 and CCL4 are primarily produced in leukocytes [156]. Other chemokines such as the major human neutrophil attractant CXCL8 or IL-8 may be induced in almost any cell type [157].

GPCRs have seven transmembrane helices with three extra and three intracellular loops, an extracellular N-terminus and intracellular C-terminus. Chemokines bind to the extracellular domain and to a pocket in the transmembrane area and the signal is transmitted to the intracellular compartment. Cells are activated by the direct coupling to G proteins or β arrestins [154,158,159]. The intracellular signaling in the chemokine receptors is related to second messengers such as calcium, cyclic adenosine monophosphate (cAMP) and GTPases (Ras and Rac). The GPCRs can also signal through β arrestins, a pathway that can regulate the receptor signal through the desensitization process [159,160]. β arrestins can block the binding to the phosphorylated G proteins and they are responsible for internalization of receptors to endosomes and degradation. Desensitization may be critical for maintaining the capacity of the cell to sense a chemoattractant gradient [161]. Multiple ACKRs, which fail to signal through the G proteins, have been reported to signal through β arrestins [154,162,163].

In total, 20 chemokine receptors are described and they are all expressed on leukocytes. Based on their functions, they can be divided into constitutive and inducible or homeostatic and inflammatory receptors. Initially, inflammatory chemokines and their receptors were only studied in the context of inflammation, but some receptors were identified as co-receptors for HIV entrance into the cell and others are associated with tumor metastasis [164,165,166,167,168]. Regarding homeostasis, the chemokine system is involved in embryogenesis, leukocyte trafficking to lymphoid organs, tissue/organ development and angiogenesis. For instance, much attention has been given to the contribution of the CXCR4 receptor to embryogenesis, hematopoiesis, and leukocyte trafficking from bone marrow. The importance of CXCR4 in this condition is critical for survival, since the deletion of CXCR4 or its ligand CXCL12 in mice is embryonically lethal [169,170]. Already at its discovery it was recognized that CXCR4 is expressed on neutrophils [171]. CXCR4 upregulated on “aging” neutrophils was shown to induce reverse migration of senescent neutrophils from the circulation to bone marrow [172]. CXCR1 and CXCR2 were the first members of the chemokine receptor family to be cloned, sharing a high degree of homology with formyl peptide receptors (FPRs) [173,174]. Inflammatory neutrophils express high levels of CXCR1 and CXCR2 on their surface once activated and the receptors and their ligands have an important role in neutrophil recruitment [175].

The atypical chemokine receptors (ACKRs) are related to classical chemokine receptors and also have seven transmembrane domains but they are not able to activate G proteins [176,177,178,179]. These receptors are expressed on leukocytes and non-hematopoietic cells. ACKRs signal through the β arrestin pathway, but they also work as scavenger receptors, since they can internalize the bound chemokines without chemotactic actions. Neutrophils express ACKR2 (or D6), a receptor for most inflammatory CC chemokines [180,181]. ACKR2 is supposed to restrict migration of CCR1 expressing neutrophils to its ligands including the highly potent CC chemokine CCL3 [177]. CCRL2, a receptor with high homology with chemokine receptors and with chemerin as identified ligand, on the other hand was shown to heterodimerize with CXCR2 and to promote neutrophil migration in mice [178,182].

### 4.5. Regulation of Chemokine-Dependent Neutrophil Recruitment

Chemokine activity can be regulated at multiple levels, including gene duplication, gene transcription and translation. Upon stimulation with PAMPs several connective tissue cells will produce the inflammatory cytokine IL-1β. IL-1β induces the production of chemokines, CXCL8 being the most potent neutrophil attracting chemokine in human. In addition, neutrophils that are recruited to the inflammatory joint may enhance the response through the secretion of additional active IL-1β further enhancing the production of CXCR1/CXCR2 ligands and neutrophil accumulation [183]. Some pre-formed chemokines are stored in endothelial cells, inside secretory granules including Weibel-Palade bodies, and are quickly released upon cell insult [184]. Once produced, chemokine activity can be regulated by binding to glycosaminoglycans on endothelial cell layers of lymph and blood vessels, by binding to and expression of GPCRs and ACKRs on cells, or by receptor-mediated synergy and antagonism among chemokines [184,185,186]. Recently, microRNAs, regulating the chemokine and chemokine receptor mRNA levels, were discovered as a novel mechanism for fine-tuning chemokine and chemokine receptor expression [187,188]. Finally, chemokines and their receptors become post-translationally modified. Chemokines can be modified post-translationally through: (1) proteolytic cleavage by enzymes such as metalloproteinases, CD26 and enzymes from pathogens [189,190,191,192]; (2) citrullination, that is the formation of citrulline by the deimination of arginine by peptidylarginine deiminases (PAD) [193,194,195]; (3) *N*-glycosylation on asparagine within an Asn-Xaa-Ser/Thr motif, or *O*-glycosylation on serine (Ser) or threonine (Thr) residues [196]; and (4) nitration, where peroxynitrite, produced during oxidative stress, can selectively oxidize and nitrate several residues, including the oxidation of histidine and the nitration of tyrosine and tryptophan [197,198,199]. Reduced or enhanced receptor affinity and chemokine activity have been reported, depending on the chemokine and on the type of posttranslational modification [189]. Most posttranslational modifications of inflammatory chemokines are dependent on proteolytic cleavage, mainly affecting the N-terminal region of the protein with highly specific proteases.

In addition to specific GPCRs, glycosaminoglycans (GAGs) play an important role in the regulation of neutrophil migration. GAGs are linear carbohydrate structures, consisting of a repeating disaccharide unit, that comprises a hexuronic acid linked to an *N*-acetyl-hexosamine that can be sulfated at different positions [200,201,202]. GAGs are negatively charged and can be divided into six groups: heparan sulfate, heparin, chondroitin sulfate, dermatan sulfate, keratan sulfate and hyaluronic acid. These sugar units can bind or attach to protein cores of proteoglycans or can be found associated with the extracellular matrix. GAGs are heterogeneous in length and composition and they can bind to a huge number of proteins. GAGs have fundamental roles in cell signaling and development, angiogenesis, tumor progression, embryogenesis, wound healing, and have anti-coagulant properties [203,204]. Interestingly, GAGs can interact directly with pathogens. Particularly related to this study, hyaluronic acid favors to increase lubrication in synovial joints. The loss of hyaluronic acid in osteoarthritic patients is associated with an increase of pain and stiffness [205].

**Figure 3:**
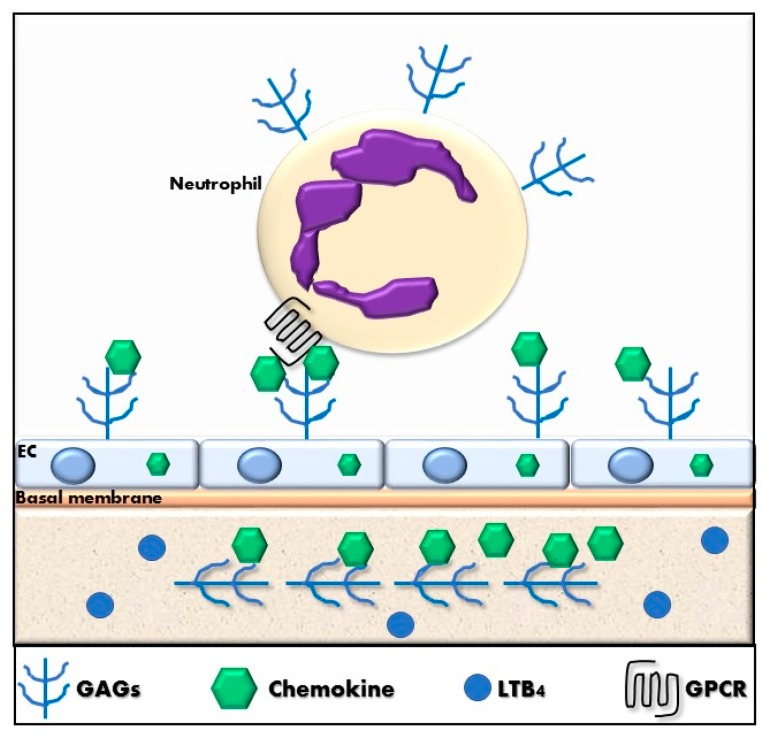
Neutrophil recruitment by chemokines and leukotriene B4. Neutrophils are recruited to the tissue through chemoattractants such as chemokines and LTB4. Chemokines bind to GAGs, which are expressed on endothelial cells and tissue. The retention of chemokines on GAGs generates a chemokine gradient that favors the binding of chemokines to their GPCR. LTB_4_ binds to specific GPCRs on the neutrophil to induce firm adhesion of the cell to the endothelium. GAGs: glycosaminoglycans, LTB_4_: leukotriene B_4_, GPCR: G protein-coupled receptor.

Each tissue produces specific GAG repertoires and cells can alter their GAG expression in response to specific stimuli or in pathologic states. GAGs are important in cell recruitment during homeostatic and inflammatory processes by their direct interaction with chemokines [202,206]. The binding of chemokines to GAGs can generate an immobilized chemokine gradient that directs cell migration, as shown in Figure 3. Cell surface immobilization of chemokines enables them to act locally rather than as paracrine molecules, and likely prevents inappropriate activation and desensitization of receptors on cells outside the region of interest for a given physiological situation [207]. Moreover, GAG expression on the leukocyte also influences the chemokine interaction with GPCRs on the same cell [208,209].

Almost all chemokines are basic proteins, often with a pI of 10 or higher, with many Arg, Lys and His residues and GAGs bind to proteins with positive charges. The epitopes for GAG binding on chemokines are described to be BBXB and BBBXXB motifs, where B represents a basic amino acid [210]. It has been shown that some chemokines act as monomers, whereas many chemokines can oligomerize and form diverse quaternary structures including dimers, tetramers and polymers, increasing the number of epitopes that bind to GAGs [211,212]. Oligomerization increases the affinity of chemokines for GAGs through an avidity effect and this interaction also stabilizes the chemokine oligomers. Moreover, oligomerization may have a dramatic effect on GAG affinity and specificity [213,214].

### 4.6. The 5-Lipoxygenase Pathway: Mechanisms of Neutrophil Recruitment and Inflammation

At the onset of inflammation, classic lipid mediators are produced, including LTB4, which activate and amplify the cardinal signs of inflammation [215]. 5-lipoxygenase (5-LO) is a main enzyme involved in the production of these lipid mediators. This enzyme is expressed in leukocytes such as neutrophils, macrophages, dendritic cells, B cells and T cells [216]. During the inflammatory process, another class of arachidonic acid (AA)-derived lipids, prostaglandins E2 and D2, induce the switch of leukotriene synthesis to pro-resolving lipid production, including lipoxin A4 (LXA4) [189,217,218]. The generation of anti-inflammatory resolvins assists in the control of the inflammatory process [219]. The synthesis of LXA4 is also dependent on 5-LO. LXA4 has an important role in the resolution of inflammation by decreasing neutrophil migration. On the other hand, LXA4 increases the recruitment of macrophages. Additionally, LXA4 increases the phagocytosis of apoptotic neutrophils by macrophages, a process named efferocytosis, to avoid tissue damage [220].

#### 4.6.1. Leukotriene B4

LTB4 is a very potent chemoattractant for neutrophils. LTB4 is produced from AA in a pathway dependent on lipoxygenases (LO) [98]. AA is a 20-carbon fatty acid that is present in all cells and it is the main eicosanoid precursor. Some stimuli such as *N*-formyl-methionyl-leucyl-phenylalanine (fMLF), CXCL8, microorganisms, phagocytic particles and damage or injury can activate phospholipases and release AA from the cell membrane [221]. In the cytosol AA can be metabolized into leukotrienes and lipoxins via a pathway dependent on LO. The main LO enzymes are 5-LO, that is expressed in leukocytes, and 12/15-LO, expressed in reticulocytes, eosinophils, immature dendritic cells (DCs), epithelial and airway cells, pancreatic islets and resident peritoneal macrophages [222]. The first step in leukotriene biosynthesis is the conversion of AA into a hydroperoxide, named 5-hydroperoxyeicosatetraenoic acid (5-HPETE), by the insertion of an oxygen at position 5. In this step the activation of 5-LO is dependent on the 5-LO activating protein (FLAP). 5-HPETE can be reduced to 5-hydroxyeicosatetraenoic acid (5-HETE) or can be converted in a 5,6-epoxide containing a conjugated triene structure, named leukotriene A4 (LTA4) by removal of a water molecule [223]. LTA4 is instable and can be converted into LTB4 by insertion of a hydroxyl group at carbon 12 by the enzyme LTA4 hydrolase. Another possibility is the conversion in leukotriene C4 (LTC4) by addition of a glutathionyl group at carbon 6 by γ-glutamyl-*S*-transferase [224]. LTB4 is produced and released within minutes by neutrophils, macrophages, and mast cells and is an important element of the immediate inflammatory response [225].

Leukotrienes bind to extracellular GPCRs, which are members of the rhodopsin-like receptors family and related to chemokine receptors. LTB4 is known to bind to two LTB4 receptors, BLT1 and BLT2 [226]. BLT1 is a 43 kDa GPCR expressed in inflammatory cells, such as neutrophils, alveolar macrophages, eosinophils, differentiated T cells, dendritic cells and osteoclasts, and has a high affinity for LTB4. The BLT2 receptor has low affinity for LTB4 and is expressed more ubiquitously [227]. BLT1 is widely related to chemotaxis. The axis LTB4/BLT1 is needed for neutrophil recruitment in arthritis and for the recruitment of neutrophils to lymph nodes during bacterial infection. On the other hand, the axis LTB4/BLT2 is involved in the generation of reactive oxygen species and can enhance wound healing [225,228,229].

#### 4.6.2. Lipoxin A4

Lipoxins can be generated by three main pathways. In the first one, AA is released from the cell membrane and one oxygen is inserted at carbon 15 by 15-LO in eosinophils, monocytes or epithelial cells, resulting in the intermediate 15S-HPETE. 15S-HPETE can be taken up by neutrophils or monocytes and converted in the 5,6-epoxytetraene by 5-LO and then is hydrolyzed by LXA4 or lipoxin B4 (LXB4) hydrolases in LXA4 and LXB4 [230,231]. The second route involves the interaction between leukocytes and platelets. The 5-LO present in leukocytes, such as neutrophils, converts AA into LTA4 as described before. The LTA4 is released and taken up by adherent platelets. These express 12-LO that converts LTA4 in LXA4 and LXB4 [232]. The third route occurs after the exogenous administration of aspirin. In this case, aspirin triggers the formation of the 15R-epimer of lipoxins, 15-epi-LXA4 and 15-epi-LXB4. These epimers carry a carbon 15 alcohol group in the R configuration. They arise from aspirin-acetylated by cyclooxygenase-2 (COX-2) and share the actions of LXA4 [233].

LXA4 binds to the GPCR receptor ALX/FPR2. ALX is expressed on leukocytes, astrocytoma cells, epithelial cells, hepatocytes, microvascular endothelial cells and neuroblastoma cells. Unlike classic GPCRs for chemoattractants that mobilize intracellular Ca^2+^ to evoke chemotaxis, lipoxins instead induce changes in the phosphorylation of proteins of the cytoskeleton, resulting in β arrestin activation [234,235]. LXA4 presents pro-resolving actions such as decreased neutrophil infiltration, increased recruitment of mononuclear cells and an increase in the uptake of apoptotic neutrophils by macrophages. LXA4 has also effects on the return of vascular permeability to normal levels [236].

Both lipids LTB4 and LXA4 have been described to be involved in articular diseases. LTB4 and 5-LO mRNA was found in synovial tissue of patients with rheumatoid arthritis [237,238]. LTB4 is also associated with pathogenesis in the collagen-induced arthritis model, the K/BxN serum transfer arthritis model [239,240,241] and the experimental model of gout [242]. LXA4 was also detected in synovial tissue of patients with rheumatoid arthritis [243]. Nonetheless, in zymosan-induced arthritis, LXA4 was related to attenuation of the disease [244]. During infection, LTB4 and LXA4 are related to clearance of pathogens and improvement of the disease. Some studies show that LTB4 has a role in the control of lung Paracoccidioidomycosis and is important for phagocytosis and killing of *Borrelia burgdorferi* [245,246]. In lung infection by *Cryptococcus neoformans* and sepsis, LXA4 is associated with the control of infection and an increase in survival [247,248]. However, in pneumosepsis induced by *Klebsiella pneumoniae*, the LXA4 in the early stage of the disease is associated with systemic infection-induced mortality and can improve survival at a late stage of the disease [249].

## 5. Dual Functions of Neutrophils during Septic Arthritis

Based on the aforementioned mechanisms of the pathology of septic arthritis, this disease is associated with severe articular damage and pain. During the immune response against *S. aureus* infection, neutrophils are the main cells recruited to the joint. As previously mentioned, neutrophils contain potent antimicrobial molecules that are important in the control of infection, including joint infections. The depletion of neutrophils prior to the systemic injection of *S. aureus* in mice impaired the bacterial control and increased mortality. Furthermore, the absence of neutrophils increased the circulating levels of pro-inflammatory cytokines and the frequency of arthritis after three days of infection, suggesting that a systemic inflammatory response against a high titer of *S. aureus* could also cause arthritis independent of the toxic effects of neutrophils [15]. Similarly, the increased concentration of neutrophils in the joint due to a photodynamic therapy applied locally improves the clearance of MRSA and decreases tissue damage [250]. These examples highlight the importance of neutrophils to control *S. aureus*-induced arthritis. However, neutrophils are associated with articular damage and pain development [251,252]. Lögters et al. identified increased NETosis in synovial fluid of septic arthritis patients, mainly patients infected with *S. aureus*, compared to noninfectious or osteoarthritic joints. Importantly, there was a positive correlation with levels of IL-6 and IL-1 in the joints [253].

The blockade of neutrophil migration and activity could be an interesting strategy to avoid excessive articular damage and pain during *S. aureus*-induced septic arthritis in mice [254,255]. Formylated peptides are potent neutrophil chemoattractants. In mice, the intravenous injection of *S. aureus* carrying a mutation that prevents the synthesis of formylated peptides decreases the accumulation of neutrophils in the joint when compared to wild type *S. aureus* injection. In mice injected with mutated bacteria, the incidence of arthritis was lower, associated with decreased synovitis and cartilage damage as compared to injection with wild type *S. aureus*. However, the bacterial load analyzed in the joints is comparable between the two bacteria at seven days after injection, suggesting that the neutrophils guided by formylated peptides are important for joint inflammation and damage [13].

The specific blockage of neutrophil recruitment in order to avoid or reduce tissue damage was also successful using CXCR1/2 inhibitors in some animal models of inflammatory articular diseases such as antigen-induced arthritis, collagen-induced arthritis, autoantibody-mediated arthritis and gout [254,255,256,257,258]. Since neutrophils have a crucial role during the infection, the blockade of these cells in septic arthritis can be protective or detrimental. CXCR2-binding chemokines are present in the joint of *Brucella mellitensis*-infected mice, guiding the recruitment of neutrophils. Neutrophil counts correlated with joint inflammation and damage. Indeed, CXCR2-deficient mice displayed delayed incidence, reduced clinical scores, and decreased swelling as compared to WT mice, although there is no difference on bacterial load between both groups [259]. We previously demonstrated in a septic arthritis model induced by the intra-articular injection of *S. aureus* that the treatment with an antagonist of CXCR1/2 starting from the beginning of the infection was able to decrease neutrophil recruitment, articular damage and hypernociception. Nonetheless, the bacterial load increased, showing that neutrophils are important for bacterial control [24]. In addition, we also evaluated if the blockage of neutrophils at a later time point of the infection could be effective. The treatment starting 3 days after the infection, partially reduced hypernociception, prevented the increase in bacterial load, but failed in inhibiting articular damage. Besides the blockage of neutrophil migration, the decrease of neutrophil activation could also be useful to control joint inflammation and damage in septic arthritis. Activated neutrophils produce hypochlorous acid (HOCl) by a reaction of myeloperoxidase with hydrogen peroxide. The accumulation of HOCl is toxic and its blockage could be beneficial to avoid excessive tissue damage. Taurine is an amino acid found abundantly in the cytosol of neutrophils and acts as scavenger of HOCl. Interestingly, the injection of taurine chloramine (a product of taurine and HOCl) in the joint at the same time of *S. aureus* significantly reduced the histopathological score, especially cartilage and bone destruction [260]. Thus, the benefits of targeting neutrophil activation or migration during septic arthritis still need to be better clarified, considering effects of different bacterial strains and disease progression. Therefore, it is clear that the neutrophils recruited to the joint in the initial phase of the infection play a role in the control of infection, but can cause articular damage. Thus the blockage of neutrophils as a potential therapy for septic arthritis needs to be carefully evaluated in order to create a balance between control of infection (with or without co-treatment with antibiotics) and induction of articular damage.

## 6. Conclusions

In most cases, the host has the ability to induce a protective inflammatory response resulting in elimination of the invading pathogen and subsequent resolution of infection. However, if the infection cannot be controlled and persists, strong activation of the immune response can cause destruction of the joint [11]. Accordingly, most of the detrimental effects of infection result from the exaggerated immune response of the host rather than from direct cytotoxicity of bacteria [6].

The role of neutrophils in the pathogenesis of septic arthritis is dual. On the one hand, they are indispensable in the early phase of disease for effective defense against bacteria and consequently for host survival. On the other hand, these leukocytes act as mediators of tissue-destructive events. The massive infiltration of neutrophils into the infected joint can contribute to cartilage and bone destruction by the release of free radicals and bacteria- and tissue-degrading enzymes, including products of the NADPH oxidase complex and proteolytic enzymes targeting collagen and/or proteoglycans. Permanent destruction of cartilage and subchondral bone loss can occur within three days after infection [11,13,15]. Elimination of neutrophils from the site of inflammation is a prerequisite for resolution of the acute inflammatory response. This implicates that prolonged presence of neutrophils at the site of inflammation can lead to persistence of the inflammatory response, associated with complications such as tissue damage [261,262].

Other factors playing a role in joint damage include: high levels of cytokines, which enhance the release of MMPs (such as MMP-2, MMP-3 and MMP-9) and other enzymes degrading collagen, and bacterial toxins and enzymes [11]. Furthermore, the inflammatory process following infection alters the synovium as well as the composition and cellular content of the synovial fluid. These changes in the synovial fluid might affect the articular cartilage, since synovial fluid is indispensable for lubrication and nutrition of the articular cartilage. Moreover, synovial fluid dynamics can be disrupted by the infectious process, leading to a fluid effusion in the joint. Subsequently, intra-articular pressure increases, preventing the supply of blood and nutrients to the joint, thereby resulting in destruction of the synovium and cartilage [2,11].

Early and effective treatment may enable resolution of the inflammatory process. In case of unsuccessful or no treatment, articular cartilage may be lost entirely, resulting in fibrous or bony joint ankylosis. When inhibition of neutrophil infiltration is considered as a treatment option, co-treatment with antibiotics will probably be essential to combine diminished tissue destruction with efficient clearance of the microorganisms.

## Figures and Tables

**Table 1:** Chemokine receptors expressed on neutrophils and their human and murine ligands ^1^.

Receptor	Human Ligand(s)	Murine Ligand(s)
CXCR1	CXCL6, CXCL8	CXCL6
CXCR2	CXCL1, CXCL2, CXCL3, CXCL5, CXCL6, CXCL7, CXCL8	CXCL1, CXCL2, CXCL3, CXCL6, CXCL7
CXCR3	CXCL4, CXCL4L1, CXCL9, CXCL10, CXCL11,	CXCL4, CXCL9, CXCL10, CXCL11
CXCR4	CXCL12	CXCL12
CCR1	CCL3, CCL3L1, CCL4L1, CCL5, CCL7, CCL8, CCL14, CCL15, CCL16, CCL23	CCL3, CCL5, CCL6, CCL7, CCL9
CCR2	CCL2, CCL7, CCL8, CCL13, CCL16	CCL2, CCL7, CCL12
CCR3	CCL3L1, CCL5, CCL7, CCL8, CCL11, CCL13, CCL15, CCL24, CCL26, CCL28	CCL5, CCL7, CCL9, CCL11, CCL14, CCL24, CCL26, CCL28
CCR5	CCL3, CCL3L1, CCL4, CCL4L1, CCL5, CCL8, CCL11, CCL14, CCL16	CCL3, CCL4, CCL5
ACKR2	Inflammatory CC chemokines	Inflammatory CC chemokines

^1^ CXCR1 and CXCR2 are highly expressed on circulating neutrophils, CXCR4 is enhanced on “aging” neutrophils and other chemokine receptors may be upregulated on neutrophils in inflamed tissues.

## Abbreviations

AA	Arachidonic acid
ACKR	Atypical chemokine receptor
DC	Dendritic cell
FPR	Formyl peptide receptor
GAG	glycosaminoglycan
GPCR	G protein-coupled receptor
HETE	hydroxyeicosatetraenoic acid
HPETE	hydroperoxyeicosatetraenoic acid
IL-	Interleukin-
LTA4	Leukotriene A4
LTB4	Leukotriene B4
LTC4	Leukotriene C4
LO	Lipoxygenase
LXA4	Lipoxin A4
MPO	myeloperoxidase
MRSA	methicillin-resistant *Staphylococcus aureus*
MSCRAMM	microbial surface component recognizing adhesive matrix molecules
NETs	neutrophil extracellular traps
PAMP	pathogen-associated molecular pattern
PGN	peptidoglycan
PRR	pattern recognition receptor
RA	rheumatoid arthritis
ROS	reactive oxygen species
*S. aureus*	*Staphylococcus aureus*
TLR	Toll-like receptor
TNF	tumor necrosis factor
